# The Self-Compassion Scale Short Form for Children Aged 8–12: Validation of the Italian Version

**DOI:** 10.3390/children12010084

**Published:** 2025-01-13

**Authors:** Valentina Levantini, Iacopo Bertacchi, Antonella Magno, Gianluca Domenico Privitera, Alessia Rinaldi, Nicoletta Zara, Pietro Muratori

**Affiliations:** 1 Department of Languages and Literatures, Communication, Education and Society, University of Udine, 33100 Udine, Italy; valentina.levantini@uniud.it; 2 Accademia di Neuropsicologia dello Sviluppo (A.N.Svi), University of Pisa, 42124 Reggio Emilia, Italy; iacopo.bertacchi@ospiti.unipi.it; 3 Azienda Sanitaria Potenza—ASP Basilicata, AIPC, 70125 Bari, Italy; antonella.magno@aspbasilicata.it; 4 Istituto Comprensivo Statale “Campanella-Sturzo”, 95121 Catania, Italy; privigia@libero.it; 5 IRCCS Fondazione Stella Maris, Scientific Institute of Child Neurology and Psychiatry, 56018 Pisa, Italy; a.rinaldi15@studenti.unipi.it; 6 Struttura Complessa di Neuropsichiatria dell’Infanzia e Adolescenza, ASL di Oristano, 09170 Oristano, Italy; nicoletta.zara@asloristano.it

**Keywords:** questionnaire, narcissism, compassion, mindfulness, youth

## Abstract

Background: Although self-compassion has been consistently linked to positive outcomes in adults and adolescents, only a few studies have explored self-compassion in children and early adolescents due to a lack of measures specifically designed for younger populations. Methods: The current study explored the psychometric properties of the Italian translation of the Self-Compassion Scale for Children (SCS-C) in a sample of 230 children (mean age = 10.52, SD = 1.33). Results: The results revealed a two-factor solution for our data, with a positive self-compassion factor and a negative self-compassion factor, which were shown to be internally consistent. The SCS-C scores correlated with the children’s mindfulness, self-esteem, and narcissistic traits in the assumed directions. Conclusions: Overall, our study preliminarily supports the reliability and validity of the Italian version of the SCS-C.

## 1. Introduction

Self-compassion (SC) is commonly described as a caring attitude toward oneself. It refers to the way individuals care for themselves during times of hardship or when encountering setbacks [1]. Neff [1] has developed a commonly used definition of SC, which consists of three elements. The first is self-kindness, which refers to being sympathetic and understanding toward oneself rather than critical and harsh. The second element is common humanity, which recognizes imperfection, difficulties, and failure as shared human experiences. The third one is mindfulness, which is described as awareness of one’s present-moment negative experience. Even though different definitions of SC have been proposed over time, in line with Buddhist conceptualizations, it is believed, e.g., [2], to encompass affective, behavioral, and cognitive components such as recognizing pain, understanding that suffering is a universal part of the human experience, empathizing with distress, tolerating the uncomfortable emotions it evokes, accepting the situation, and taking action to ease it. Many studies have highlighted links between SC and several positive outcomes in adults and youths. Extensive research has shown that SC is positively associated with well-being, life satisfaction, self-esteem, and positive affects [3,4,5,6]. At the same time, studies have found that SC is negatively associated with depression, anxiety, and stress [1,4,7]. Overall, the findings emphasize the benefits of being compassionate toward oneself.

Self-compassion is believed to begin at an early age, and its development is highly related to parenting and attachment security [8,9]. Despite being present throughout development [1], only limited research is currently available on SC during late childhood and early adolescence. This is connected to a scarcity of measures of SC specifically developed or adapted for younger populations.

The Self-Compassion Scale (SCS) [1] is a measure that is commonly used to assess self-compassion in adults through six factors: self-kindness, self-judgment, common humanity, isolation, mindfulness, and over-identification. Several studies have investigated its psychometric properties; however, the factor structure remains a topic of ongoing debate, with the definite number of factors being one of the main concerns [10].

Recently, Neff et al. [4] developed and evaluated the psychometric properties of the Self-Compassion Scale-Youth version (SCS-Y) in a series of studies conducted with samples of middle school students. The results revealed a bifactor structure, with a general self-compassion factor and six specific subscale scores (i.e., self-kindness, self-judgment, common humanity, isolation, mindfulness, and over-identification). Internal consistency, test–retest reliability, and construct validity (e.g., associations with mindfulness, life satisfaction, and resilience) were supported.

Sutton et al. [5] evaluated the psychometric properties of the Self-Compassion Scale for Children (SCS-C) in a large sample of 406 children aged 8 to 12 years. The SCS-C is a 12-item self-report measure adapted from the Self-Compassion Scale–Short Form proposed by Raes et al. [11]. The findings of the study pointed toward a two-factor structure for the SCS-C, with two different subscales, named positive self-compassion (PSC), which refers to self-kindness, mindfulness, and common humanity, and negative self-compassion (NSC), which refers to self-judgment, over-identification, and isolation. Both factors showed good internal consistency. The children’s scores on the PSC and NSC subscales were also correlated with measures of mindfulness, self-concept, well-being, and empathic skills, as hypothesized by the study’s authors, providing further support for the scale’s structure.

Preadolescence represents a pivotal developmental phase characterized by several physical, emotional, and cognitive changes that can often pose challenges to the well-being of young individuals. Given the potential vulnerability during this stage of development, identifying and studying skills and traits that might positively impact children’s mental health and their later outcomes is a matter of critical importance. One such skill, self-compassion, has been widely acknowledged as a significant protective factor, e.g., [5]; however, as previously mentioned, there remains a notable gap in the research dedicated to developing and validating measures of SC specifically tailored to assess this construct in youths. Addressing this gap is highly relevant for advancing our understanding of SC’s role during this formative stage and informing interventions to promote resilience and emotional well-being.

Based on this, this study aimed to investigate the psychometric properties of the Italian translation of the Self-Compassion Scale for Children. Specifically, we tested the SCS-C factor structure and the internal consistency of its scores. We then tested the convergent and discriminant validity of the SCS-C scores by examining their relationships with measures of mindfulness, self-esteem, and narcissistic traits. The latter construct was selected because previous studies found that it was negatively related to SC [12,13] and usually related to adverse outcomes in youths, e.g., [14]. We hypothesized that SC would be positively associated with children’s mindfulness and self-esteem levels but negatively associated with narcissistic traits [4,5,12].

## 2. Materials and Methods

### 2.1. Participants and Procedure

The sample included 230 children (123 boys and 107 girls) aged 8 to 11 years (mean age = 10.52, SD = 1.33). The participants were recruited during the 2022/2023 academic year; this protocol was included in projects delivered in the school setting. Before completing the questionnaires, parents were asked to provide written informed consent to participate. The procedures were carried out in accordance with the 1964 Helsinki Declaration and its later amendments. The “Commissione Benessere” of the Istituto Comprensivo Statale “Campanella-Sturzo”, Catania (Italy), revised and approved this study in October 2022.

### 2.2. Measures

Self-compassion. The participants were asked to complete the Self-Compassion Scale for Children [5]. This scale was translated into Italian in collaboration with Prof. Sutton. The items assessed each of the six components outlined in Neff’s definition of self-compassion [1]: self-kindness (“I try to be kind towards those things about myself I don’t like.”), self-judgment (“I am hard on myself about my own flaws/weaknesses.”), common humanity (“When I fail at something, I try to remember that everybody fails sometimes too.”), isolation (“When I fail at something that’s important to me, I feel like I’m all alone.”), mindfulness (“When something upsets me I try to stay calm.”), and over-identification (“When I’m feeling sad, I can’t stop thinking about everything that’s wrong.”). The items were rated on a 5-point Likert scale. The items measuring self-judgment, isolation, and over-identification (1, 4, 8, 9, 11, and 12) were reverse-scored.

Mindfulness. The children completed the Child and Adolescent Mindfulness Measure (CAMM) [15,16], a 10-item measure assessing present-moment awareness and nonjudgmental, nonavoidant responses to thoughts and feelings (e.g., “I keep myself busy so I don’t notice my thoughts or feelings”; “I push away thoughts that I don’t like”; and “I think about things that have happened in the past instead of thinking about things that are happening right now.”). Their answers were rated using a 5-point Likert scale. In this sample, Cronbach’s α was 0.700.

Self-esteem. Global self-esteem was evaluated using Rosenberg’s Self-Esteem Scale [17,18], a 10-item self-report measure (e.g., “On the whole, I am satisfied with myself”; “I am able to do things as well as most other people”; and “I take a positive attitude toward myself.”). The items were answered using a 4-point Likert scale. In this sample, Cronbach’s α was 0.818.

Narcissistic traits. The children’s narcissistic traits were assessed with the Childhood Narcissism Scale (CNS) [14,19]. The CNS is a self-report measure that includes 10 items rated on a 4-point Likert scale (e.g., “It often happens that other kids get the compliments that I actually deserve”; “I am very good at making other people believe what I want them to believe”; and “I like to think about how incredibly nice I am.”). In this sample, Cronbach’s α was 0.757.

### 2.3. Data Analysis

Statistical analysis was performed using IBM SPSS Statistics for Windows, Version 26.0 and RStudio. We conducted confirmatory factor analysis (CFA) with the RStudio package *lavaan* (version 0.6-1.1240) using a robust maximum-likelihood (MLR) estimator to test the factor structure of the SCS-C. We tested six competing models: (a) a one-factor model [20]; (b) a two-factor model including positive self-compassion (positively worded items: 2, 3, 5, 6, 7, and 10) and negative self-compassion (negatively worded items: 1, 4, 8, 9, 11, and 12) factors [5,21]; (c) a six-factor model including self-kindness, self-judgment, common humanity, isolation, mindfulness, and over-identification factors [22]; (d) a second-order two-factor model including the two specific factors reported in model *b* and a second-order self-compassion factor [23]; (e) a second-order six-factor model including the six specific factors reported in model *c* and a second-order self-compassion factor; and (f) a bifactor model encompassing two specific factors (positive self-compassion and negative self-compassion) and a general self-compassion factor [24]. The goodness of fit of the models was tested using the scaled chi-square (χ^2^) and several robust indices, including the Comparative Fit Index (CFI), the Tucker–Lewis Index (TLI), the Root-Mean-Square Error of Approximation (RMSEA) with 90% confidence intervals (CIs), and the Standardized Root-Mean-Square Residual (SRMR). CFI and TLI values ≥ 0.90 were interpreted as acceptable, and values ≥ 0.95 were interpreted as excellent. SRMR and RMSEA values ≤ 0.08 were interpreted as acceptable, and values ≤0.06 were interpreted as excellent [25,26].

The internal consistency of the SCS-S was assessed using Cronbach’s α and the mean inter-item correlations (MICs). Finally, we calculated bootstrapped zero-order correlations using IBM SPSS Statistics to test the convergent and discriminant validity. Bootstrapped analyses were conducted using 1000 bootstrapped samples and 95% bias-corrected accelerated confidence intervals (95% BCa CIs). Convergent validity was evaluated by examining the associations between the SCS-C scores and the measures of mindfulness and self-esteem [5], while discriminant validity was tested by examining the associations between the SCS-C scores and the children’s levels of narcissistic traits [12].

## 3. Results

### 3.1. Factor Structure

The fit indices of the competing models are reported in Table 1. The models involving six factors (i.e., models *c* and *e*) showed convergence issues. A review of the parameter estimates revealed negative residual variances for item 6. Negative residual variances might be indicative of estimation problems and often occur when a model is overparameterized (e.g., there are too many factors and/or too few indicators for each factor) or when a hypothesized model does not adequately represent the underlying structure of the data. In models *c* and *e*, each factor was measured by only two indicators, which met the minimum requirement for model identification but provided limited information for parameter estimation. Despite these issues, modifications were not made to address the negative variances. Removing item 6, and as a consequence, the associated self-kindness factor, would have compromised the theoretical integrity of the model. Therefore, the fit indices of these models are not reported.

The bifactor model (model *f*) showed the best fit indices. However, further investigation of the factor loadings highlighted some issues with the factor structure. Items 1, 4, 8, 9, 11, and 12 did not significantly load into their specific factors. Therefore, the bifactor structure was not deemed adequate for our data.

Models *b* and *d* showed almost overlapping fit indices. Since the TLI and RMSEA were slightly better for model *b* and more parsimonious models were preferred, the two-factor model was judged to have the best fit.

As the fit indices of model *b* were below the suggested cut-offs, we searched for possible signs of model misspecification by exploring the factor loadings and modification indices (MIs). We found that item 2 *(*“I try to be kind towards those things about myself I don’t like”) did not significantly load into the positive self-compassion factor (estimate: 0.175, *p* = 0.065). Moreover, the MIs (>22) suggested strong covariances between the error terms of items 11 (“I am hard on myself about my own flaws and weaknesses”) and 12 (“I get frustrated or upset about the things about myself I don’t like”) and between items 5 (“When I fail at something, I try to remember that everybody fails sometimes too”) and 10 (“When I feel like I’m not good enough at something, I try to remind myself that everyone feels that way sometimes”). After making these modifications, the model’s goodness of fit improved, suggesting an excellent fit (see Table 2 for the standardized factor loadings).

### 3.2. Reliability

Cronbach’s α was 0.807 and the MIC was 0.412 for negative self-compassion, while α was 0.727 and the MIC was 0.347 for positive self-compassion. Overall, the SCS scores showed acceptable-to-good internal consistency.

### 3.3. Validity

As shown in Table 3, the results showed low-to-moderate positive associations between positive self-compassion and mindfulness and self-esteem measures. Negative self-compassion, instead, was strongly and positively associated with both mindfulness and self-esteem measures. Finally, positive self-compassion was not related to narcissistic traits, while negative self-compassion was negatively and significantly associated with them.

## 4. Discussion

For the first time, the current study investigated the psychometric properties of the Italian translation of the Self-Compassion Scale for Children. Confirmatory factor analysis (CFA) supported a two-factor solution for our data, with one factor composed of all the positively worded items (PSC) and the other composed of the negatively worded ones (NSC). However, the results revealed that item 2 (“I try to be kind towards those things about myself I don’t like”) did not significantly load into the PSC subscale, and it was removed. It is possible that children, especially younger ones, might find this item hard to understand because of how it is articulated; a revision of its formulation could be considered, at least in the Italian translation.

CFA also highlighted strong covariances between the error terms of items 11 (“I am hard on myself about my own flaws and weaknesses”) and 12 (“I get frustrated or upset about the things about myself I don’t like”) and items 5 (“When I fail at something, I try to remember that everybody fails sometimes too”) and 10 (“When I feel like I’m not good enough at something, I try to remind myself that everyone feels that way sometimes”), which might suggest content overlap, redundancy, or a common source of error. In both pairs, the items belong to the same subscales (i.e., PSC for items 5 and 10 and NSC for items 11 and 12), and even if worded differently, their contents might appear similar, making it reasonable for children to interpret and rate these items in the same way.

In the scientific literature, there is an ongoing and significant debate regarding the factor structure of the SCS and its abbreviated version, the SCS-SF, which was the focus of the present study. Following Neff’s [1] indications, most studies have relied on SCS or SCS-SF total scores as indicators of global self-compassion. However, this approach has been subject to criticism, with some researchers arguing that relying solely on a single total score may not effectively represent the underlying construct and that two different scores should be preferred. Specifically, concerns have been raised about the inclusion of negatively worded items, which some believe may tap into psychological constructs highly related to psychopathology (e.g., self-criticism and self-focused rumination) rather than the self-kindness, common humanity, and mindfulness of Neff’s model, e.g., [27,28].

Although our results are highly preliminary, they indicate that two correlated factors—and consequently, two distinct scores—may offer a better fit for the data. We observed comparable fits for both the first-order and second-order two-factor models. Nonetheless, according to model selection principles, it is generally recommended to opt for the more parsimonious model. Moreover, PSC and NSC were shown to be moderately correlated; this suggests that while the two factors are related, they still maintain enough independence to justify treating them as separate components within the model. This separation is important for accurately capturing the distinct dimensions of self-compassion and providing a clearer understanding of its underlying structure. Additionally, the two factors were internally consistent, as shown by the Cronbach’s alphas and mean inter-item correlations.

These findings are in line with other studies endorsing the use of two separate factors for the SCS [5,21,29,30]. However, given the relatively small sample size—which may have influenced the assessment of the more complex models in the confirmatory factor analysis—and the age range of our participants—which was different from previous studies—more research is needed to further explore the factor structure of the Italian translation of the SCS-C.

The correlation analysis supported the validity of the PSC and NSC scores. Specifically, both SCS-C subscales were significantly and positively correlated with the children’s self-reported mindfulness, with NSC showing a much stronger association. This evidence is consistent with previous studies [5] and is particularly relevant since mindfulness, which is intended to indicate awareness of one own distress, is considered a core element of SC, and SC can be interpreted as a product of global mindfulness [1,5].

As hypothesized, PSC and NSC were also positively associated with self-esteem. Despite some dissimilarities [31], self-esteem and SC share some important aspects. Both are self-related constructs that reflect a positive attitude toward oneself and are believed to be beneficial for individuals’ well-being and mental health; therefore, it is not surprising that they are positively correlated [32]. Moreover, as Muris and Otgaar [32] argued, people who positively value themselves and have solid “true” self-esteem might be more likely to exhibit self-compassion during challenging times. This relationship underscores the idea that individuals with healthy self-esteem are better equipped to treat themselves with kindness and understanding when faced with adversity.

Finally, correlations showed that PSC was unrelated to narcissistic traits, while NSC was negatively linked to them. This was in line with previous research showing that SC is not related to [33] or negatively correlated with narcissism [12]. From a theoretical perspective, indeed, SC is differentiated from and does not promote narcissism, self-absorption, or self-centeredness because of common humanity, which should convey a sense of belonging in contrast to isolation [1,4]. Narcissism is often associated with a critical and judgmental inner dialogue; this internal conflict can contribute to various psychological challenges, including emotional distress and interpersonal difficulties. In this regard, our findings open up interesting hypotheses for intervention models targeting narcissistic traits in children. These traits, when left unaddressed, can lead to a range of negative outcomes, such as internalizing problems and externalizing behaviors, which are observed in both community and clinical samples [14,34]. Given the potential for early interventions to mitigate these adverse consequences, our research could inform strategies designed to help children with narcissistic tendencies develop healthier self-views and improve emotional regulation, ultimately preventing or reducing the impact of these traits on their mental health and overall well-being.

The results of the current study should be interpreted in light of some limitations, including the small sample size, the cross-sectional and correlational design, and the sole use of self-report data. Future studies should enroll larger samples, assess other measures relevant to SC (e.g., well-being and internalizing symptoms), and incorporate a multimethod assessment approach (e.g., parent-report tools).

## 5. Conclusions

Overall, the current study provides support for the validity of SCS-C scores, paving the way for future studies on SC during childhood and early adolescence. SC is a protective factor that has gathered substantial consideration within the context of mental health problems in adults [35]. A growing body of evidence shows that research results in children and adolescents align with what has been found in the adult literature, further emphasizing that SC is a protective factor against psychopathology, e.g., [36,37]. This has significant implications for preventive and treatment programs. Indeed, promoting SC and self-soothing skills might enhance youth mental health and well-being, as shown by intervention programs that specifically focus on improving SC [38,39].

## Figures and Tables

**Table 1:** The fit indices of the competing models.

	Scaled χ^2^	Robust TLI	Robust CFI	Robust RMSEA	SRMR
One-factor model (model *a*)	227.729 (54), *p* < 0.001	0.647	0.711	0.126 [0.109, 0.143]	0.107
Two-factor model (model *b*)	135.321 (53), *p* < 0.001	0.829	0.863	0.087 [0.069, 0.106]	0.087
Second-order two-factor model (model *d*)	132.768 (52), *p* < 0.001	0.826	0.863	0.088 [0.070, 0.107]	0.087
Bifactor model (model *f*)	60.543 (42), *p* = 0.032	0.952	0.970	0.046 [0.014, 0.071]	0.044
Modified two-factor model	52.985 (41), *p* = 0.099	0.971	0.978	0.038 [0.001, 0.066]	0.054

**Table 2:** The standardized factor loadings of the modified two-factor model.

Items	Factor 1 Positive Self-Compassion	Factor 2 Negative Self-Compassion
Item 3	0.688 [0.575, 0.801] *	-
Item 5	0.588 [0.459, 0.717] *	-
Item 6	0.703 [0.601, 0.805] *	-
Item 7	0.500 [0.368, 0.631] *	-
Item 10	0.441 [0.287, 0.595] *	-
Item 1 ^R^	-	0.612 [0.494, 0.730] *
Item 4 ^R^	-	0.629 [0.522, 0.737] *
Item 8 ^R^	-	0.634 [0.525, 0.742] *
Item 9 ^R^	-	0.661 [0.550, 0.773] *
Item 11 ^R^	-	0.635 [0.512, 0.757] *
Item 12 ^R^	-	0.678 [0.560, 0.796] *

Note: Correlation between Factor 1 and Factor 2: *r* = 0.551 [0.375, 0.727], *p* < 0.001. R = reverse-coded; * *p* < 0.001.

**Table 3:** Bootstrapped correlation coefficients of SCS-C scores and child-reported mindfulness, self-esteem, and narcissistic traits with 95% BCa confidence intervals.

Items	1	2	3	4	5
1. Positive SC	-				
2. Negative SC	0.391 ** [0.248, 0.524]	-			
3. CAMM	0.134 * [0.001, 0.274]	0.647 ** [0.559,0.724]	-		
4. Self-Esteem	0.481 ** [0.347,0.600]	0.648 ** [0.568, 0.722]	0.509 ** [0.403, 0.610]	-	
5. Narcissistic Traits	−0.076 [−0.221, 0.071]	−0.213 ** [−0.338, −0.080]	−0.151 * [−0.280, −0.022]	0.041 [−0.96, 0.174]	-

Note: SC: self-compassion; CAMM: Child and Adolescent Mindfulness Measure. * *p* ≤ 0.05; ** *p* ≤ 0.01.

## Data Availability

The data are available from the corresponding author upon reasonable request.
